# *Houttuynia cordata* Extract Ameliorates Bladder Damage and Improves Bladder Symptoms via Anti-Inflammatory Effect in Rats with Interstitial Cystitis

**DOI:** 10.1155/2020/9026901

**Published:** 2020-10-09

**Authors:** Wenbiao Li, Fei Yang, Hailun Zhan, Bolong Liu, Jiarong Cai, Yun Luo, Xiangfu Zhou

**Affiliations:** Department of Urology, The Third Affiliated Hospital of Sun Yat-sen University, Guangzhou, Guangdong 510630, China

## Abstract

The mechanism of interstitial cystitis/bladder pain syndrome (IC/BPS) remains unclear to date, but reports showed that bladder inflammation and increasing number of activating mast cells in bladder tissues were common in patients with IC/BPS. *Houttuynia cordata* is widely used in Chinese traditional medicine, and its function of anti-inflammation has been proved. The purpose of this study was to investigate the efficacy and possible mechanisms of the *Houttuynia cordata* (HC) extract in the treatment of interstitial cystitis/bladder pain syndrome (IC/BPS). In the current study, a total of 30 adult female rats were randomly divided into three groups: sham group (*n* = 10), cyclophosphamide + saline (CYP + NS) group (*n* = 10), and cyclophosphamide + *Houttuynia cordata* extract (CYP + HC) group (*n* = 10). The animal model of IC/BPS was induced with cyclophosphamide (75 mg/kg, intraperitoneal injection, once every 3 days for 10 days) in the CYP + NS group and CYP + HC group, and sham rats received a volume-matched injection of saline. After anesthesia with urethane (0.8 g/kg, intraperitoneal injection), intravesical administration of either saline (1 ml) or *Houttuynia cordata* extract (1 ml, 2 g/ml) was continued once per day for a week in the CYP + NS group and CYP + HC group, respectively. Subsequently, urinary frequency, nociceptive behaviors, cystometry, bladder weight, histological changes, and cytokine (IL-6, IL-8, TNF-*α*) concentration were evaluated and compared among the three groups. Variables including inflammatory grade, mast cell number, proportion of activated mast cells, bladder weight, cytokine concentration of bladder homogenates, and frequency of urination significantly increased in the CYP + NS group compared with the sham group (*P* < 0.01) and CYP + HC group (*P* < 0.01). Besides, compared with the CYP + NS group, longer intercontraction interval, bigger bladder capacity, higher nociceptive threshold, fewer number of mast cells, and lower proportion of activated mast cells were found in the CYP + HC group (*P* < 0.01). Our study demonstrated that the *Houttuynia cordata* extract can effectively inhibit mast cell proliferation and activation and downregulate proinflammatory cytokine in a rat model of IC/BPS induced with cyclophosphamide and might be potentially valuable for the treatment of IC/BPS.

## 1. Introduction

Interstitial cystitis/bladder pain syndrome (IC/BPS) is a chronic bladder inflammatory disease, characterized by urinary urgency, frequency, nocturia, and suprapubic pain, in the absence of infection or other identiﬁable causes [1]. The prevalence of IC/BPS is increasing, and to date there is no cure. The unpleasant symptoms persist or relapse frequently and cost lots for treatment [2]. Although the underlying mechanism of IC/BPS remains unclear and the consensus on the pathophysiology is lacking, bladder inflammation appears to be common in many IC/BPS patients and increased number of activating mast cells in the bladder tissues were detected [3]. The inflammatory mediators such as interleukin-6 (IL-6), interleukin-8 (IL-8), and tumor necrosis factor-*α* (TNF-*α*) released from mast cells play important roles in the inflammation and bladder-associated pelvic pain of IC/BPS [4]. Thus, the suppression of mast cell proliferation and control of inﬂammatory reaction may be an important aspect for treating IC/BPS.

*Houttuynia cordata* (HC) is widely used in Chinese traditional medicine. Previous studies suggested that HC contains many polyphenolic components which possessed many pharmacological functions, including antioxidant, antitumor, antiallergic, and anti-inflammatory properties [5–7]. Its function of anti-inflammation had been proved, and it has been used for treating various inflammatory diseases such as suppuration, chronic bronchitis, pneumonia, and pleurisy [8–10]. As for the mechanism, a previous study reported that HC could inhibit mast cell activation effectively [11]. In addition, some reports demonstrated that HC suppresses the release of inflammatory mediators such as IL-6, IL-8, and TNF-*α* in LPS-treated RAW 264.7 cells and HMC-1 human mast cells [12–14].

Based on the previous studies, the aim of the present study is to investigate whether the *Houttuynia cordata* extract is able to restore bladder damage and improve bladder symptoms in the rat model of IC/BPS and try to find out the related mechanisms.

## 2. Materials and Methods

### 2.1. Preparation of *Houttuynia cordata* Extract

*Houttuynia cordata* extract (HC) (Xinfeng Pharmaceutical Co., Ltd., affiliated company of Guangdong Pharmaceutical University, Guangdong, China) was prepared as follows: fresh *Houttuynia cordata* (2000 g) was exposed to steam distillation. The forerunning liquid (200 ml) was collected for redistillation, and then sodium chloride (7 g) and polysorbate (5 g) were added to the redistilled liquid (1000 ml).

### 2.2. Animal Groups and Treatment Protocol

Thirty adult female Sprague-Dawley rats (200–250 g in weight) were treated under a protocol approved by the Sun Yat-sen University Institutional Animal Care and Use Committee (IACUC-2013-0803). The rats were randomly assigned to three groups (10 rats in each group): (1) sham group, (2) cystitis induced by cyclophosphamide (CYP) and treated with saline (CYP + NS group), and (3) cystitis induced by cyclophosphamide and treated with the *Houttuynia cordata* extract (CYP + HC group). Chronic cystitis was induced with cyclophosphamide as described previously (75 mg/kg; intraperitoneal injection, once every 3 days for 10 days) [16], and rats in the sham group received a volume-matched injection of saline. Then, after anesthesia with urethane (0.8 g/kg, intraperitoneal injection), rats in the CYP + HC group received treatment of intravesical administration of the HC extract (1 ml, 2 g/ml, once per day for 1 week), while rats in the sham group and CYP + NS group received volume-matched intravesical administration of saline (1 ml, once per day for 1 week).

### 2.3. Urinary Frequency

The day after the final treatment, all rats were tested for urinary frequency and low-volume voids. All rats received intraperitoneal injection of methylene blue (20 mg/kg, once) 5 hours before the test, and water was taken away from the rats 2 hours before the test. Then, all rats were placed in cages individually with filter paper bedding. After 30 minutes, the filter papers were collected and numbers of total urine spots and low volume spots (diameter shorter than 5 mm) were counted [17]. Urine spots were blue due to the methylene blue and were easy to be identified.

### 2.4. Nociceptive Behavior

Von Frey monofilament testing was proved to be a reliable method in nociceptive behavior measurement by several reports. Nociception testing in our study was carried out based on the previous studies [18–20]. In detail, treatment of each rat was hidden to the observer. Each rat was placed individually in a clear plastic box which was divided into 6 small chambers with a metal grid floor and allowed to acclimatize to the environment for about 30 min. Mechanical stimulation was performed using von Frey monofilaments. The filaments were applied to the lower abdominal area between the anus and external urethral oriﬁce using 8 different von Frey ﬁlaments (Stoelting, Kiel, WI, USA) with increasing bending forces of 1 g, 2 g, 4 g, 8 g, 15 g, and 26 g. Each ﬁlament was applied 10 times for 1-2 s once at intervals of 5–10 s. Care was taken not to stimulate the same point twice in succession to avoid learning or sensitization. Scoring of nociceptive behavior was deﬁned as follows: 0, no response; 1, licking or biting of the external urethral opening and/or the surrounding area, leaving the position, bending of the trunk, raising the upper half of the body, thrashing limbs, and jumping. Once the total scores are greater than 5 in ten times stimulation, the very force was defined as the nociceptive threshold of the rat.

### 2.5. Cystometry

All rats were anesthetized with urethane (0.8 g/kg, intraperitoneal injection). Cystometry was carried out at a rate of 6 ml/h through a urethral PE 50 catheter. The catheter was connected to a pressure transducer and syringe pump through a three-way stopcock. The bladder capacity, intercontraction intervals, and bladder compliance were recorded.

### 2.6. Histopathology Evaluation

The bladders of all rats were harvested and weighed. Half of each bladder was made into bladder homogenate for the detection of cytokine. The rest of the bladder tissue was fixed in buffered formalin (10%) for 48 h, embedded in paraffin, cut into 4 *μ*m transverse sections, and stained with hematoxylin-eosin and toluidine blue. Bladder inﬂammation was evaluated using a four-point scoring system in the sections stained with hematoxylin-eosin: 0, morphologically unremarkable with no or minimal inﬂammation or epithelial changes; 1, mild inﬂammatory inﬁltrate within the lamina propria, accompanied by mild chronic edema, hemorrhage or urothelial changes, and ﬁbrosis scattered in the lamina propria; 2, moderate inﬂammatory inﬁltrate in the lamina propria and focal extension of the inﬂammation into the muscularis propria, accompanied by moderate chronic edema, hemorrhage, urothelial changes, and ﬁbrosis diffused in the lamina propria; 3, severe inﬂammation in the lamina propria and muscularis propria in association with other signiﬁcant ﬁndings, such as urothelial ulceration, severe chronic edema, hemorrhage, and diffused ﬁbrosis through the bladder [20].

The sections stained with toluidine blue were used for mast cell count, and measurements in each section were represented by the average of 5 random high-power ﬁelds (×200). Then, by turning to higher power fields (×400), the proportion of activated mast cells of the 5 abovementioned fields was observed.

Both bladder inflammation and mast cell count were assessed by two pathologists in a blinded fashion.

### 2.7. Cytokine Analyses

Bladder homogenates were prepared at a concentration of 100 mg tissue/ml PBS. All samples were centrifuged at 3,000 rpm for 20 min. The supernatants were collected, and the levels of inflammatory cytokines (IL-6, IL-8, and TNF-*α*) were measured by a commercially available rat ELISA kit (Guangzhou Biological Technology Co., Ltd, China) on a microplate reader (Bio-Rad, USA).The absorbance was read at 450 nm. Standard curves for each cytokine were generated using the reference cytokines supplied with the kits.

### 2.8. Statistical Analysis

Statistical analysis was performed with SPSS 18.0, and the Mann–Whitney *U*-test was used to assess nonparametric data such as inflammatory grade and nociceptive threshold among three groups. Quantitative data were expressed as mean ± standard deviation (SD) and evaluated using one-way ANOVA with Bonferroni correction. All tests were 2-sided, and *P* < 0.05 was considered statistically significant.

## 3. Results

One rat in the sham group, two rats in the CYP + NS group, and two rats in the CYP + HC group were dead because of anesthesia accident, and the experiment was performed successfully in the remaining rats.

### 3.1. Histopathology Evaluation

**Figure 1:**
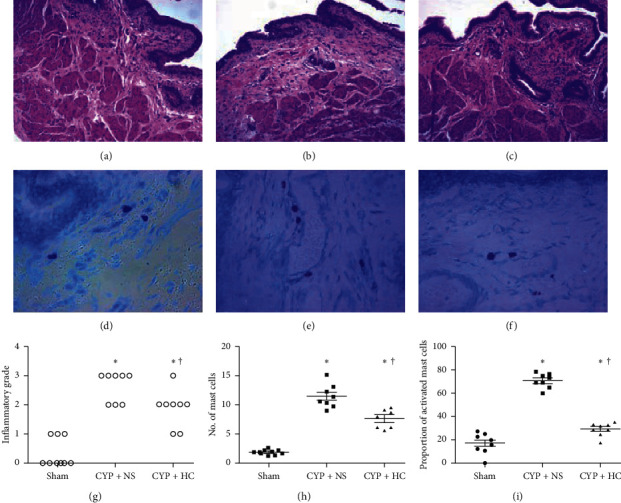
*Houttuynia cordata* extract restored bladder damage and inhibited mast cell proliferation and activation. (a) Hematoxylin-eosin staining (×100) of the sham group. (b) Hematoxylin-eosin staining (×100) of the CYP + NS group: inﬂammatory cell inﬁltration, urothelial injury, submucosal edema, and hemorrhage were observed obviously. (c) Hematoxylin-eosin staining (×100) of the CYP + HC group, inflammatory reaction of bladder tissue improved. (d) Toluidine blue staining (×400) of the sham group: few mast cells and no activated mast cells were observed.(e) Toluidine blue staining (×400) of the CYP + NS group, large numbers of mast cells were observed and most of them were activated mast cells. (f) Toluidine blue staining (×400) of the CYP + HC group: fewer number of mast cells and activated mast cells were observed. (g) Inflammatory grade of the CYP + HC group was significantly lower than that of the CYP + NS group. (h) Number of mast cells of the CYP + HC group was fewer than that of the CYP + NS group. (i) Proportion of activated mast cells of the CYP + HC group was fewer than that of the CYP + NS group. ^*∗*^*P* < 0.01 vs. the sham group; ^†^*P* < 0.01 vs. the CYP + NS group.

Administration of cyclophosphamide to rats significantly increased the urinary bladder weight compared with the sham group (117.9 ± 13.5 mg vs. 83.2 ± 3.4 mg, *P* < 0.01), which was inhibited by the treatment with the HC extract (117.9 ± 13.5 mg vs. 101.6 ± 6.7 mg, *P* < 0.01). As for the bladder tissues of rats in the CYP + NS group, inﬂammatory changes including inﬂammatory cell inﬁltration, urothelial injury, submucosal edema, and hemorrhage were observed and the number of mast cells and the proportion of activated mast cell were significantly higher compared with the sham group (*P* < 0.01). While in the CYP + HC group, the inﬂammatory grade was lower and the number of mast cells and proportion of activated mast cells in bladder tissues were far fewer than those of the CYP + NS group (*P* < 0.011; Figure 1).

### 3.2. Cytokine Concentration Assay

**Figure 2:**
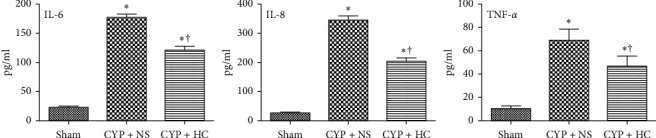
*Houttuynia cordata* extract decreased the release of cytokines. Concentration of cytokines (IL-6, IL-8, and TNF-*α*) of bladder homogenates signiﬁcantly decreased after HC-treatment, when compared with the CYP + NS group.^*∗*^*P* < 0.01 vs. the sham group; ^†^*P* < 0.01 vs. the CYP + NS group.

As shown in Figure 2, the concentrations of cytokines (IL-6, IL-8, and TNF-*α*) of bladder homogenates were signiﬁcantly increased in the CYP + NS group compared with the sham group, respectively (*P* < 0.01), and concentrations of cytokines in the CYP + HC group were significantly lower than those of the CYP + NS group (*P* < 0.01).

### 3.3. Functional Evaluation

**Figure 3:**
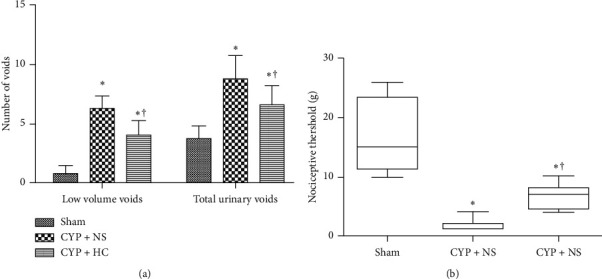
*Houttuynia cordata* extract improved bladder symptoms (urinary frequency and bladder pain) of rat models. (a) The number of low urine voids and total urinary voids was less in the CYP + HC group, compared with the CYP + NS group. (b) Nociceptive threshold of the CYP + HC group was signiﬁcantly higher than that of the CYP + NS group. ^*∗*^*P* < 0.01 vs. the sham group; ^†^*P* < 0.01 vs. the CYP + NS group; ^††^*P* < 0.05 vs. the CYP + NS group.

Compared with the sham group, increased urinary frequency and low-volume voids were observed in cyclophosphamide-treated rats (*P* < 0.01), which can be decreased by HC extract treatment (Figure 3(a)). In addition, nociceptive threshold decreased significantly in cyclophosphamide-induced rats (*P* < 0.01) and restored significantly after treatment with the HC extract (*P* < 0.01) (Figure 3(b)). Cystometric parameters including intercontraction intervals, bladder capacity, and bladder compliance signiﬁcantly decreased in the CYP + NS group compared with the sham group (*P* < 0.01), and these parameters were improved by HC extract treatment (*P* < 0.01, Table 1).

## 4. Discussion

Cyclophosphamide-induced cystitis is well known to be a reliable rat model of IC/BPS. In this study, cyclophosphamide injection to rats caused symptoms characterized by urinary frequency, urinary urgency, pelvic pain, bladder inflammation, and mast cell proliferation, similar to those in human with IC/BPS.

IC/BPS is a nonbacterial disease associated with urinary frequency, urgency, and bladder pain, and the treatment for patients with IC/BPS is still challenging for urologists. Although the exact etiology and pathogenesis of IC/BPS remain unknown, there are studies suggesting that inflammation may play a key role in the pathogenesis of this disease manifested by the increased concentration of inflammatory mediators [4, 21, 22]. Besides, the elevated number and activation of mast cells in the patients with IC have been reported by many studies [23] and mast cells have been shown to participate in bladder inflammation via releasing cytokines and chemokines [4, 24]. Cytokines and chemokines such as TNF-*α*, IL-6, and IL-8 have a crucial role in the bladder inflammation [4], and the increased levels of these cytokines have been reported in IC/BPS patients and the experimental cystitis model [4, 25]. IL-6, which is released by mast cells, is elevated in both urine and serum of IC/BPS patients, especially in patients with severe inflammation, and positively associated with pain scores [26]. Furthermore, Erickson et al. found an association between IL-6 and symptom severity in IC/BPS patients [27]. TNF-*α*, which is secreted by activated mast cells, causes expression of adhesion factors on vascular endothelial cells and accumulates white blood cells, resulting in an inflammatory response [28]. IL-8 induces inflammatory response by acting as a chemotactic factor for neutrophils, eosinophils, and T lymphocytes [29, 30]. In the present study, a greater number of mast cells and a higher expression of proinflammatory cytokines were detected in rats treated with cyclophosphamide, which were associated with urothelial injury, submucosal edema, hemorrhage, and inflammatory cell infiltration in the bladder. These findings were consistent with previous studies, further suggesting that mast cells and proinflammatory cytokines may be involved in the pathogenesis of interstitial cystitis. Some previous reports indicated that bladder inflammation increases afferent sensitivity and nociception through the upregulated expression of nociceptive receptors on nerve terminals and production of neurotransmitters, contributing to the symptoms of urinary frequency, urgency, and pelvic pain in IC/BPS patients [25, 31, 31]. Therefore, mediating bladder inflammation through suppressing mast cell proliferation and inhibiting release of proinflammatory mediators may be an effective therapy for IC/BPS.

*Houttuynia cordata* is commonly used in Chinese traditional medicine; different solvent extracts of HC have been shown to be effective against inflammation. The *Houttuynia cordata* extract used in the present study was commercially available and its constitution was similar. *Houttuynia cordata* has been used for treating various inflammatory diseases such as suppuration, chronic bronchitis, pneumonia, and pleurisy [8, 13]. However, to date, we did not find any reports on the use of *Houttuynia cordata* in clinical and experimental studies involving the therapy of interstitial cystitis/bladder pain syndrome. Recent studies reported that *Houttuynia cordata* could effectively suppress the activation of mast cells and inhibit release of inflammatory mediators through various ways [5, 6, 8, 11–14]. Li et al. further indicated that *Houttuynia cordata* extract could inhibit the activation of mast cells by blocking calcium uptake in mast cells or increasing the cAMP level in mast cells [11]. Besides, Kim et al. demonstrated that *Houttuynia cordata* supercritical extract decreased serum level of IL-6 and TNF-*α* in the rat inflammation model by inhibiting both TNF-*α*-NO and COX-2-PGE_2_ signaling pathways [10]. In addition, some reports found that *Houttuynia cordata* extract suppresses the production of TNF-*α*, IL-6, and PGE2 in LPS-treated RAW 264.7 cells by inhibition of nuclear factor-kB (NF-kB) activation and MAPK signaling pathway [13]. Similarly, Lee et al. also detected that *Houttuynia cordata* inhibits the production of proinflammatory cytokines including TNF-*α*, IL-6, and IL-8 through inhibition of the NF-*κ*B signaling pathway in HMC-1 human mast cells [14]. Based on the previous studies, we tried to use *Houttuynia cordata* to treat IC/BPS. Intravenous or intramuscular injection of the *Houttuynia cordata* extract has the risk of allergy or even anaphylactic shock. Also, intravesical perfusion is an important treatment approach for bladder diseases, especially for IC/BPS. The *Houttuynia cordata* extract was delivered to the bladder cures inflammation directly, avoiding the risk of adverse reactions effectively. In the current study, the mast cell number and concentration of inflammatory mediators (TNF-*α*, IL-6, and IL-8) of bladder homogenates significantly decreased in the CYP + HC group compared with the CYP + NS group, and bladder inflammation also improved significantly, suggesting that the *Houttuynia cordata* extract had an anti-inflammatory effect in cystitis induced by cyclophosphamide. Furthermore, bladder symptoms including urinary frequency and pelvic pain induced by cyclophosphamide were also improved significantly after treatment with *Houttuynia cordata*, as evidenced by the less number of urine spots, shorter intercontraction intervals, and higher nociceptive threshold, due to the improvement of bladder inflammation. It has been reported that mast cells were associated with detrusor fibrosis and further decreased bladder compliance in patients diagnosed with IC/BPS [26], and in the present study, bladder compliance was significantly increased in HC extract-treated rats than saline-treated rats, which was consistent with the previous findings.

## 5. Conclusion

Our study firstly reported the efficacy of *Houttuynia cordata* in the therapy of interstitial cystitis/bladder pain syndrome. The result showed that the *Houttuynia cordata* extract could effectively restore bladder damage and improve symptoms of urinary frequency and pelvic pain in a rat model of IC/BPS induced by cyclophosphamide, via inhibiting mast cell proliferation and activation and downregulating expression of proinflammatory mediators. Therefore, we speculated that the *Houttuynia cordata* extract had potential to be an economical and effective therapy for patients with IC/BPS.

## Figures and Tables

**Table 1:** Comparison of cystometric parameters of three groups.

Groups	ICI (S)	BC-1 (ml)	BC-2 (ml/cmH_2_O)
Sham	623.7 ± 43.6	1.27 ± 0.08	0.0425 ± 0.0039
CYP + NS	192.9 ± 19.5^*∗*^	0.62 ± 0.08^*∗*^	0.0253 ± 0.0053^*∗*^
CYP + HC	489.6 ± 16.8^*∗*†^	0.96 ± 0.07^*∗*†^	0.0372 ± 0.0056^*∗*†^

ICI, intercontraction interval; BC-1, bladder capacity; BC-2, bladder compliance; ^*∗*^*P* < 0.01 vs. the sham group; ^†^*P* < 0.01 vs. the CYP + NS group.

## Data Availability

The datasets used and/or analyzed during this study are available from the corresponding author on reasonable request.
